# Nursing experiences of COVID‐19 outbreak in Iran: A qualitative study

**DOI:** 10.1002/nop2.604

**Published:** 2020-08-19

**Authors:** Ahmad Kalateh Sadati, Leila Zarei, Saeed Shahabi, Seyed Taghi Heydari, Vajihe Taheri, Razieh Jiriaei, Najme Ebrahimzade, Kamran Bagheri Lankarani

**Affiliations:** ^1^ Department of Social Sciences Yazd University Yazd Iran; ^2^ Health Policy Research Cente, Institute of Heath Shiraz University of Medical Sciences Shiraz Iran; ^3^ Qazvin University of Medical Science Qazvin Iran; ^4^ Arak University of Medical Sciences Arak Iran

**Keywords:** coronavirus outbreak, COVID‐19, nursing experience, sacrificial commitment, stigma

## Abstract

**Aim:**

The global outbreak of coronavirus in 2020 was considered as a serious risk for healthcare providers, especially nurses. This study aimed to investigate nurses’ perceptions and experiences of COVID‐19 outbreak in Iran.

**Design:**

This thematic analysis study was conducted in March 2020.

**Method:**

Semi‐structured interviews were conducted with 24 nurses in Qazvin, Arak, Shiraz and Kashan cities of Iran.

**Results:**

It was found out that all the participants had faced a mysterious world created by the virus. No one had clear understanding of the new virus and knew how to tackle with such a virus. In this case, the main experiences were related to defected preparedness, the worst perceived risk, family protection, social stigma and sacrificial commitment. Urgent preparedness of facilities in such outbreaks is inevitable. Accordingly, psycho‐social support of nurses and their families and strengthening their sacrificial commitments are proposed in these conditions.

## 1 INTRODUCTION

Respiratory diseases annually cause about 5 million deaths worldwide; however, an epidemic is likely to increase this number significantly (Zumla & Niederman, 2020). Recently, these diseases have been associated with viruses such as coronavirus (Lai, Shih, Ko, Tang, & Hsueh, 2020). A novel coronavirus, designated as severe acute respiratory syndrome coronavirus 2 (SARS‐CoV‐2) causing COVID‐19, was first diagnosed in Wuhan, China, at the end of 2019 (Munster, Koopmans, van Doremalen, van Riel, & de Wit, 2020). The COVID‐19 quickly spread throughout the world (Wang et al., 2020) so that the world health organization (WHO) declared the outbreak a global pandemic on 11 March 2020 (WHO).The spread of the disease has been rapid as such it has become a worldwide pandemic (Sadati, Lankarani, & Lankarani, 2020). Since there is currently no valid treatment for this disease, the main approach is symptomatic and supportive treatments, which include keeping vital signs, preserving oxygen saturation and blood pressure and treating complications such as secondary infections or organ failure. To this end, it is also important to prevent its infection or further spread by individuals travelling to viral epidemic sites through measuring their body temperature and recommending them to have self‐care for 14 days (Wu, Chen, & Chan, 2020).

Nurses, as the first line of care providers of hospitalized patients, play a fundamental role in the treatment and prevention of the ascending trend of the disease. Such pandemics lead to difficult and unpredictable conditions (Barnett et al., 2012). Nurses' perception of their willingness to respond to uncertainty and security in a pandemic and to provide the right services for the needs of the community during this period has impacts on the health of the community. Studies have shown that the lower risk perception of nursing services poses greater courage and willingness among service providers and vice versa (Basta, Edwards, & Schulte, 2009; Devnani, 2012).

## 2 BACKGROUND

During the outbreak of Middle East respiratory syndrome (MERS), it was found that the perception of social stigmatization, level of agreement with infection control measures and perceived risk were three factors affecting emergency nurses’ ethical problems in Korea (Choi & Kim, 2018). A study on nurses in 2015 showed that the nurses’ intention to be involved in nursing during the outbreak of MERS was related to their previous outbreak experiences (Oh et al., 2017). A qualitative study in South Korea during MERS outbreak showed that the nurses’ main experiences in this regard were feeling burnout owing to the heavy workload, relying on personal protective equipment for safety, being busy catching up with the new guidelines related to MERS and caring for suspected or infected patients with caution (Kang, Son, Chae, & Corte, 2018). Nursing experiences during Ebola outbreak in Central Africa also revealed the following main themes: (a) lack of protective gear, basic equipment and other resources necessary for care provision; (b) stigmatization; and (c) exceptional commitment of nursing profession (Hewlett & Hewlett, 2005). Regarding the nurses’ experiences during MERS outbreak in South Korea, the other study indicated that the main experienced problems were anxiety, social isolation, unprepared treatment environment, the burdensome of MERS care process and reflection on the steps to be further prepared for new infectious diseases (Kim, 2017). A phenomenological study among Australian nurses in H1N1 Influenza pandemic also revealed that personal protective equipment, infection control procedures, fear of contracting and transmitting the disease, adequate staff in the intensive care units, new roles for staff, morale levels, training for extracorporeal membrane oxygenation and patient care challenges were the most frequently reported challenges of the nurses (Corley, Hammond, & Fraser, 2010). Moreover, according to MERS experiences, nurses may suffer from mental health problems during an epidemic of a novel infectious disease (16). A recent study on the medical staff also noted that they experience anxiety and stress during COVID‐19 outbreak (Huang, Han, Luo, Ren, & Zhou, 2020).

Obviously, like all countries affected by the epidemic, COVID‐19 outbreak was one of the most serious social issues in Iran. While health policymakers have tried to control the incidence of this epidemic across the country through controlling all cases, especially the infected students residing in China, the report issued on February 20 made a social shock. The outbreak was first reported in Qom, and the early reports indicate that the virus carrier seemed to be a merchant who travelled between Qom and Wuhan, China, where COVID‐19 appeared. Evidently, the hospital and nursing staff were at the forefront of fighting against COVID‐19. Although the reports of the outbreak have been regularly presented, the hospital readiness for the outbreak has not been met since, on 2 April 2020, Iran ranked sixth regarding the highest frequency of COVID‐19 deaths after Italy, China, Spain, United States and France and seventh regarding the highest frequency of SARS‐CoV‐2 cases across the world (3,036 COVID‐19 deaths in Iran with 47,593 confirmed infections). The statistics along with doubts about the capability of hospitals in face of the epidemic posed a kind of social anxiety.

There is no study on Iranian nurses' experiences in dealing with epidemics. Given the importance and position of nurses in the epidemics, especially COVID‐19 epidemic, the present study was to investigate the perceptions and experiences of nurses in the face of coronavirus outbreaks.

## 3 METHODS

### 3.1 Design

The present study was qualitative study conducted in March 2020 in Shiraz, Arak, Kashan and Qazvin hospitals, Iran. At this time, Iran's bed was at the height of the first peak of the COVID‐19 epidemic. The most important focus of social attention was hospitals and medical staff, especially nurses. The choice of different cities was based on access to research samples.

### 3.2 Setting and sample

The study participants encompassed nurses who worked at hospitals specified for COVID‐19 treatment and the ones who were working in hospitals, known as supportive hospitals. With the continuation of the outbreak, all the hospitals in Iran, however, had to accept COVID‐19 patients as such each hospital was designated a COVID ward.

Purposive sampling technique was used to select the respondents. After providing the nurses with some explanations about the research objectives, the ones who were willing and agreed to participate were included. Interviews were conducted with respondents during their leisure time or at the end of their shifts. Based on the saturation criteria, the study participants encompassed 24 individuals from different cities, including Shiraz (*N* = 1), Arak (*N* = 6), Kashan(*N* = 1) and Qazvin(*N* = 16). The criterion sampling was finalized when data saturation was achieved.

### 3.3 Procedures

The interviews were conduced using an interview guide inclusion open‐ended questions:

- Please explain how you felt about getting prepared for COVID‐19 at the time of its outbreak?
- What concerns did you have regarding working with a confirmed or suspected COVID‐19 patient?
- What problems did you have in the ward or the hospital?

If the participants mentioned a specific point about their perception and experience of COVID‐19 exposure, the interviewer would ask them to provide further explanations. Interviews were conducted in hospitals (13 cases), home (3 cases) and call interviews (8 cases). On average, interviews lasted twenty minutes to 45 min. All interviews were conducted in Persian. The participants were Persian and familiar with the Persian language. The interviews were recorded digitally with the consent of the respondents and then transcribed. The interviews were conducted by two MA and a PhD holders familiar with the qualitative studies.

Since this article was based on a sociological study, the codes of the American Sociological Association‐*n* (Association, 2009) were applied throughout different stages of this research to observe ethical principles. Participants consent was one of the basic principles of present research ethics. Also, observing the anonymity of the participants, honesty in data analysis and presentation of results were other ethical principles that were considered in the present study. Data transcribed by a MA social worker.

### 3.4 Data analysis

Afterwards, the data were transcribed and analysed using inductive and deductive thematic analysis to extract some themes from the data. In this study, we used Braun and Clark's (Braun & Clarke, 2006) method with regard to the following six steps manually as what follows:

- Familiarize with the data, in this stage, by reading the statements several times, an attempt was made to get acquainted with the whole subject and the experience of the nurses.
- In the second stage, we assigned initial codes to the data to describe the content.
- Then, the codes were reviewed and patterns or sub‐themes were explored.
- In the forth stage, we reviewed the themes according to the codes and statements.
- Then, we defined and name themes; and
- finally, the report of nursing experiences facing with COVID‐19 was produced.

According to Braun and Clark, we have a constant moving back and forth for the entire data set, the coded extracts of the data and the analysis of the data (Braun & Clarke, 2006). In fact, there was a critical reflexivity approach in data analysis process.

Basically, trustworthiness is a main factor in any qualitative research. According to Lincoln and Guba's approach, the following four criteria promote trustworthiness (a) credibility (in preference to internal validity), (b) transferability (in preference to external validity/generalizability), (c) dependability (in preference to reliability) and (d) confirmability (Lincoln & Guba, 1986). Credibility was established using the participants’ observation and member checking. In terms of transferability, there were explicit connections with the cultural and social contexts, where the data collection was performed. For dependability, a researcher, not involved in the data collection and data analysis processes, examined these processes and the research results and confirmed them. Audit trial and critical reflexivity were also conducted to consider the conformability of the study. Using member checking, strict adherence to research steps, honesty in analysis and presentation of results were part of the research project.

This study was approved by the ethics committee of Shiraz University of Medical Sciences (IR.SUMS.REC.1395.S1249). We received written consent form from all participants in prior to the interview sessions. In addition, all participants were allowed to exit from the study at any stage voluntarily and all verbatim transcription were saved anonymously.

## 4 RESULTS

The findings showed that the nurses faced an unexpected situation and they had no experience and skill to deal with such an epidemic. They had received no relevant training during their studies. Most importantly, both the medical team and the physicians lacked the knowledge and skills required to deal with the disease. In fact, coronavirus had created an unknown and mysterious world, which was not based on the scientific principles of exposure to such a virus. As Zari said: *"Well, look, this was a situation we experienced for the first time in the hospital in this way. We had previously heard that the virus was spreading in China, but we didn't think that we were supposed to face it directly as well. When I heard that we had the virus in our hospital, I was shocked and overwhelmed with a sense of fear and anxiety because neither me nor our colleagues had sufficient knowledge and information about it*." The basic medicine and nursing principles only address the basic exposure and treatment methods and preventive care and personal protection principles. Like in the other countries, the diagnostic tools were not sufficient in Iran and the transmission ways of the disease and the treatment protocol were not fully known. Scientists and health policymakers just emphasized on protection and prevention. In this situation, the study participants experienced the following themes: defected preparedness, the worst perceived risk, family protection, social stigma and sacrificial commitment.

### 4.1 Defected preparedness

The community suddenly confronted with the corona, which consequently led a management shock. Since the Ministry of Health had succeeded in controlling HN1 flu a few months ago—December 2019, the health care staff and nurses had the same belief regarding the coronavirus disease. The sudden announcement of the corona outbreak in Iran put hospitals in a critical condition. Although a few hospitals were first introduced as the ones providing the treatment for this disease, all the hospitals were under the crisis. At the first stage of the outbreak, there was no acceptable preparedness in hospitals. Nurses experienced the lack of protective facilities and equipment. Although the coronavirus patients were not admitted to all hospitals, the suspected patients may had referred to the other hospitals, as Sahar from Qazvin said, "*We have to be so safe. It's unclear if the patient is coming is infected or not, can we respond to him/her just based on a simple temperature?"* Under these circumstances, all the hospitals were involved in the issues of personal protective equipment:

I always thought that the equipment would be perfect in the face of such a crisis. However, under the limitation of required prerequisites, you have to work in this environment with the least precision. (Marzie, Qazvin)

In the first step, all nurses requested the protections and put an additional pressure on the hospital managers:

I believed that N95 mask is not needed for everyone. I tell my staffs there is no need to even mask for an ordinary patient, but they do not agree. In this situation, no one trusts anything. (a head nurse, Shiraz)

### 4.2 The worst perceived risk

The unknown disease and, consequently, the patterns of identification and care make the exposure to infectious diseases risky. The most important concerns among the nurses were the extent of disease transmission, diagnosis, treatment, mortality rate and complications. Most of the participants reported a feeling of anxiety due to the lack of any scientific approach to the disease:

"When we were told that our hospital was going to have a corona ward in Arak, I didn't understand the crisis and couldn't figure out what was going to happen to us." (Zahra, Arak)

This ambiguity made the nurse fail to understand the risk. The diagnostic complexity of the disease was also one of the main concerns among the participants. The nurses do not know whether or not the new case is infected as they have no previous experience and have received no relevant training:

Now, it seems that you are going through a dark room, you don't know anything and it's stressful. You don't know anything until the light comes on. This is what we feel in the face of Coronavirus. (Mohammed, Qazvin)

Another point leading to anxiety is care delivery. The main question is who is infected? With each new patient being admitted, there is a growing uncertainty about whether or not he or she is infected. This is mostly due to the level of protection required for the nurses to deal with a patient. Most of the participants were concerned with not knowing if the new patient was infected or suspected. Some had experiences of working with patients who later became the infected:

Dr. Alavi was admitted for respiratory distress in our ward for a week. He used to talk to us using a cell phone. There was a close connection between the staff and him. Although he often wore a mask, it was later revealed that he was infected. (Sara, Arak)

The other source of stress was that the participants did not how much personal protection was needed. The ambiguity of the unknown disease and the uncertainty of whether or not the patient is a corona case, further enhance the level of stress with another ambiguity, that is self‐protection. In other words, the ambiguity was how much personal protection was needed and whether wearing a mask, glow and gun sufficed, or whether it required further self‐protection coverage:

I was anxious due to the ambiguity of personal protection. A doctor comes with masks and gloves and another one puts on no mask or a glove (Ali Qazvin)

One of the main causes of anxiety was to hear some news about the nurses' being infected and dead. During the first week of the epidemic, the news about the deaths of two young nurses created serious anxiety among nurses.

### 4.3 Family protection

For all the participants, a constant anxiety was the virus transmission to their families. Many participants were concerned about their families whether or they had a child, an elderly, or a person with chronic illnesses in their families:

"The more concern I had was for my family. Among my colleges, there were those whose families had a serious illness and were more concerned." (Zahra, Arak)

Even if there was no one with high risk condition, they were still worried about transmitting the disease from the hospital to their relatives. Under this condition, they were either trying to provide maximum protection or some kind of self‐quarantine:

I had three little kids at home. What could I do? I was very worried. I had to change my clothes three times. After finishing the shift in my laundry room at hospital. When I got into my car and when I got home, I was very bored and stressful. (male supervisor, Kashan)

On the other hand, since it was assumed that the disease could affect young person's asymptomatically, they concerned that they could be carriers of the disease unintentionally. Many of the participants were trying to create some sort of physical distancing between themselves and their families. Sending family members to other places such as parents or siblings’ houses, quarantining in the family and separating themselves from the family were common possible strategies for the participant's strategies. Some participants presented different types of self‐quarantine:

At the other side of the yard, we have a warehouse, I told my mom to clean up there for me to quarantine myself (Nurse, Qazvin)

f separation was not possible, this distancing would be observed in their interpersonal relationships at home, separation the bedroom, dishes and other things:

In the house, I took all my dishes away from my family and even slept away from my sister (Zahra, Qazvin)

### 4.4 Social stigma

During the coronavirus outbreak, being hospital staff means being a carrier of virus. This leads to a special behaviour towards nurses. Although the participants have not much time to be present in the social space during this period, some of them had experiences of stigma. The separation of family members from nurses has also been accompanied by a social stigma. In other words, they have had experienced obvious or latent stigma. One of the obvious stigma was experienced by Mahin's children:

My kids run away from me. They say you have Corona and I get stress, I tell myself not to get sick. They put me away. My kids run away from me. They give me something while keeping off (Mahin, Qazvin)

Samira is one of the participants affected by coronavirus and during 15 days of quarantine, her brother did not visit her. She said: "*My brother could have met me while keeping off, but he didn't come and I was upset*." If the family adopt such an approach towards the infected, there is obviously an experience of social stigma. When we were conducting this research, one of our research colleagues said that one of the medical team had told him that, despite having long conversations on cyberspace and on the phone, their friends and family members wanted to leave them early when they had visited them face to face. They thought that the nurses were carriers and there was a possibility of virus transmission:

Tow of our colleague were going to the hospital. One of them got in a taxi and the driver asked him where she was going, When she told him to get her to the hospital, he asked her to get off. The same happened to another colleague when a driver, after knowing her destination –our hospital‐ did not allow her to get in the taxi (a head Nurse, Shiraz)

### 4.5 Sacrificial commitment

Sacrificial commitment was another finding of the present study. When one is faced with the worst unpredictable situations having the highest risks, different excuses can be provided for absenteeism. You can quit your job on the pretext of taking care of your family, illness or any other reason, especially in a COVID‐19 outbreak. However, all the participants reflected the highest professional commitment. One of our participants was the Supervisor of Kashan Hospital. He had three children and had been in a home self‐quarantine for a week because of being infected by the disease. His quarantine was in the worst emotional condition. His children knew that their father was on the top floor of the building and insisted on visiting their father; however, he could not visit them. The day we interviewed him, he said he felt better and he had a shift tomorrow night on 19 March 2020 and he was admitted on the morning of the day due to fever and respiratory distress. This reflects the highest ethical and professional commitment among the participants. Our other two participants in Shiraz and Qazvin also returned to work after an initial recovery:

Look, if I don't want to come, who will come. Who will care for people? These are our citizens. We have a professional commitment and responsibility (Zahra, Arak)

Fateme said about his father response, when she said that she was to quit her job:

When I told my father not to go to work, he was very upset and he acted angrily. He said you are nurse, now people need you, you should go, now that a national crisis

Accordingly, the nurses’ families encouraged them to continue the provision of nursing care.

## 5 DISCUSSION

During the difficult and challenging period of the outbreaks, the significant role of nurses is more highlighted than ever before. The results of the present study showed that nurses and hospitals were not fully prepared for COVID‐19 epidemic, from the nurses’ perspectives. This lack of preparedness results from the lack of knowledge about the disease, as it is still unknown, on the one hand and the lake of necessary protective facilities, on the other hand. In this regard, the unknown nature of the disease also caused more ambiguity in the provision of nursing services. In this situation, the nurses perceived the highest risk. They did not know whether they were working with a patient being infected or suspected or the one with no relevant problem. On the other hand, the stress of transferring the disease to the family member made the nurses have a kind of self‐quarantine. They tried in different ways to keep their distance from their families. In some cases, there was self‐quarantine for all the nurses, especially the infected nurses. The prevalence of coronavirus and social anxiety caused a kind of stigma for nurses. This stigma ranged from family members to colleagues and general public. Finally, the present study detected a type of extra‐commitment beyond the usual day‐to‐day responsibilities of the nurses. They served under the most stressful conditions on a border between death and life.

Defected preparedness was a theme detected in this study, which was mostly related to the lack of protection equipment and facilities in hospitals. This findings is in agreement with those of the other studies during Ebola (Hewlett & Hewlett, 2005) and MERS outbreaks (Kim, 2017). Although preparedness is a part of management and is inevitable in outbreaks (Devnani, 2012), Iran suffer from some barriers to have access to the health equipment due to unfair sanctions. As Takian et al. noted, it is a shame that, besides the lives lost in this unprecedented event, extreme sanctions limit access to necessary materials and consequently kill even more Iranian individuals (Takian, Raoofi, & Kazempour‐Ardebili, 2020). Each country is likely to face different forms of this epidemic in the future. There is a global need to remove any unfair sanctions, especially in the health domain, against countries around the world.

One of the detected themes was the worst perceived risk, which was consistent with the findings of other studies on previous outbreaks (Park, Lee, Park, & Choi, 2018). This consistency is caused by the complexity of the disease as an unknown phenomenon. For example, the finding is in line with those of two other studies (Choi & Kim, 2018) (Kang et al., 2018). In Australia during the pick of the pandemic, there was a perceived lack of firm recommendations and guidelines on what specific prevention was required (Corley et al., 2010). During COVID‐19 epidemic, it was revealed that the incidence of anxiety and stress disorder was high among medical staff (Huang et al., 2020).

Social stigma was the other theme, which is in line of MERS‐CoV outbreak, indicating that both stigma and hardiness exert direct effects on the nurses’ mental health (Park et al., 2018). As Choi and Kim noted, the nurses’ ethical problems were mostly influenced by the perception of social stigmatization (Choi & Kim, 2018). In addition, Hewlett and Hewlett found that nurses had the experiences of stigmatization during Ebola outbreak (Kim, 2017). Accordingly, it seems that all medical teams are exposed to social stigma, which is more severe during the global outbreaks.

Regarding the last theme "the participants’ sacrificial commitment," it is in line with the findings of another study, introduced as exceptional commitment (Hewlett & Hewlett, 2005). In addition, a systematic review showed that the belief in duty is one factor affecting one's willingness to work during an influenza public health emergency (Devnani, 2012). It should be mentioned that nurses' actions in Iran seem to go beyond professional commitment, as a fundamental obligation based on the values of the social system, to which they are consciously or unconsciously committed. Much of these social values lie in Iran's religious values, the ones establishing a creative connection between religious and human concepts and being highlighted during social crises. As Ahmadi et al. showed, the spiritual development of Iranian nurses is related to religious obligations, commitment to ethics and commitment to law (Davoodvand, Abbaszadeh, Ahmadi, 2016). Recognizing and reinforcing these values and introducing them to educational and ethical systems are also recommended.

Generally, nurses need more social supports in these outbreaks and, as it is proposed, a public consciousness is also required to encourage healthcare workers (Choi & Kim, 2018; Kang et al., 2018). As one of the main findings of the present study, the nurses had not previously experienced of such epidemics; however, the authors of this article found valuable experiences coming from MERS epidemics in Saudi Arabia and Southeast Asia. These experiences could, in addition to being presented in the form of scientific papers, be available to nurses in all countries in a different way. It is suggested that the nurses' experiences in these epidemics be transferred to a section of nursing textbooks and teach in nursing schools. These experiences can provide an acceptable guideline for nurses all around the world to deal with the epidemics more effectively as it is also a valuable experience for nursing and hospital managers. Further training of psychological skills among medical staff, especially for female nurses, is also recommended (Huang et al., 2020).

## 6 CONCLUSION

Despite the difficult conditions posed by COVID‐19 in most countries in the world in general and for healthcare workers in particular, urgent preparedness of facilities in such outbreaks is inevitable. Accordingly, psycho‐social support of nurses and their families and strengthening their sacrificial commitments are proposed in these conditions. Hence, special attention should be paid to supportive policies to reduce job burnout in this group. After going through this difficult stage, such a supportive approach under emergency condition can be considered or revised as a foundation for dealing with future emergencies.

## 7 LIMITATION

The study also had several limitations. First, some samples were reluctant to participate in the study due to working conditions and time constraints. Second, the interviewer's presence in the hospital setting was very difficult due to the restrictions imposed after the COVID‐19 outbreak. Third, since only 24 nurses were involved in the study and the interviews were conducted in only four cities in Iran, the generalizability of our findings is limited. Fourth, given that some of the interviews were conducted at the end of the nurses' shift, their fatigue may have a negative impact on their participation. However, the research team sought to reduce these limitations by pursuing them consistently and conducting several interviews in leisure time.

## AUTHORS' CONTRIBUTIONS

AKS and ST: Study design, data analysis, interpretation of the results and manuscript drafting. VT, RJ and NE: Data analysis and interpretation of the results. AKS, LZ and SSH: Interpretation of the results and manuscript drafting. KBL: Interpretation of the results and study design. All authors confirmed the final version for submission.

## Data Availability

The data sets used and/or analysed during the current study are available from the corresponding author upon reasonable request.
